# Utilization of Crab Shell Waste for Value-Added Bioplastics by *Pseudomonas*-Based Microbial Cell Factories

**DOI:** 10.3390/ijms26062543

**Published:** 2025-03-12

**Authors:** Xiaofen Song, Hansheng Wei, Yueyue Zhou, Weiwei Song, Ce Shi, Changkao Mu, Chunlin Wang, Xiaopeng Wang

**Affiliations:** 1 Marine Economic Research Center, Donghai Academy, Ningbo University, No. 169, Qixing South Road, Meishan Port District, Beilun District, Ningbo 315000, China; songxiaofen1721@163.com (X.S.); hanshengwei2002@163.com (H.W.); songweiwei@nbu.edu.cn (W.S.); shice@nbu.edu.cn (C.S.); muchangkao@nbu.edu.cn (C.M.); wangchunlin@nbu.edu.cn (C.W.); 2 Key Laboratory of Aquacultral Biotechnology, Chinese Ministry of Education, Ningbo University, No. 169, Qixing South Road, Meishan Port District, Beilun District, Ningbo 315000, China

**Keywords:** crab shell waste, *Pseudomonas putida*, mcl-PHA, metabolic engineering

## Abstract

With the development of the aquatic products processing industry, 6–8 million tons of shrimp and crab shell waste are produced globally annually, but, due to the lack of high-value conversion technology, crab shells are often discarded in large quantities as a by-product of processing. *Pseudomonas*-based microbial cell factories are capable of biosynthesis of high-value products using a wide range of substrates; however, there is currently no reliable fermentation model for producing high-value chemicals using crab shell waste by *Pseudomonas* strains. In this study, we first explored the culture conditions of shell fermentation using KT2440 through single-factor and orthogonal experiments, and the optimized fermentation parameters obtained are given as follows: a temperature of 30 °C, fermentation time of 42 h, substrate solid–liquid ratio of 7%, and rotational speed of 200 rpm. After optimization, the maximum cell growth was increased by 64.39% from 350.67 × 10^8^ CFU/mL to 576.44 × 10^8^ CFU/mL. Combined with engineering modification, two engineered strains, KT_+IV_ and KT_+lasBT_, expressing exogenous proteases, were obtained, and the maximum growth was increased from 316.44 × 10^8^ CFU/mL to 1268.44 × 10^8^ CFU/mL and 616.89 × 10^8^ CFU/mL, which were 300.84% and 94.94% higher, respectively. In addition, the engineered strain KT_+NtrcT-D55E_, which regulates nitrogen metabolism, was obtained, and the accumulation of intracellular polyhydroxy fatty acid esters (PHA) was increased from 20.00 mg/L to 78.58 mg/L, which was a significant increase of 292.93% relative to the control group. This study provides a theoretical basis and technical support for the high-value utilization of shrimp and crab shell resources and the development of environmentally friendly bioproducts.

## 1. Introduction

In recent years, along with the booming development of the global aquatic products processing industry, a large amount of shrimp and crab shell waste (CSW) was generated every year, with the annual production of shrimp and CSW of approximately 6–8 million tons globally [1]. CSW is rich in nutrients, including proteins [2], chitin [3], chitosan [4], astaxanthin [5], etc., and simple low-value applications of these wastes mainly include composting and making feed. However, they have the potential to produce high-value products and are low-cost, stable, and abundant in supply, so their high-value utilization has become a focus of research. At present, only a small portion of CSW is used to extract chitin [4], chitosan [4], astaxanthin, and other substances [5], and these utilization methods do not give full play to its value, for example, in the process of preparing chitin from CSW, the abundant CSW proteins are often discarded. So far, due to the lack of high-value conversion technology, most of the CSW in the industry have been discarded as by-products, which not only leads to a waste of resources but also hurts the environment.

*Pseudomonas* has a strong metabolic ability to utilize a variety of organic substances as carbon and energy sources and has become a current research hotspot in the field of organic waste refining for high-value conversion [6]. Some *Pseudomonas* can produce antibiotics, such as *P. aeruginosa* [7,8]. Meanwhile, *Pseudomonas* can also secrete various enzymes, such as lipase and protease [9]. In addition, many studies have shown that *Pseudomonas* can also be used for the synthesis of polyhydroxyalkanoates (PHA) such as *P. otitis* [10], *P. hydrogenovora* [11], *P. putida* [12,13], *P. aeruginosa* [14,15], and *P. stutzeri* [16]. Among them, *P. putida* KT2440 (hereinafter “KT2440”) is widely distributed in soil, plant roots, and aqueous environments and can degrade a wide range of organic compounds [17]. Strain KT2440 is also frequently used as a heterologous host in synthetic biology and is an excellent candidate for various industrial applications due to its ability to efficiently produce a wide range of valuable compounds such as aromatic compounds, non-ribosomal peptides, and PHA [18], making it a commonly used model strain in environmental research.

PHA, also known as bioplastic, is an intracellular polyester produced by microbial cells and used as their energy reserve [19], it has excellent mechanical properties, biocompatibility, and biodegradability, and has a broad application prospect in the fields of food packaging [20], biomedical materials [21], etc. However, one of the challenges hindering the commercialization of PHA is the high cost of substrate, which is about three times more than that of polypropylene of the same mass [22]. Currently used substrates for PHA production, such as corn syrup, sucrose, and vegetable oils, have shown varying degrees of productivity [23]. Corn syrup and sucrose are widely utilized due to their high carbon content and ease of assimilation by microbial strains. At the same time, vegetable oils are favored for their high lipid content, which can significantly enhance PHA yields. However, these conventional substrates contribute to high production costs, with substrate expenses accounting for more than 40% of the total cost [23]. Moreover, their use raises ethical and sustainability concerns as they compete directly with the global food supply chain [23]. In contrast, CSW represents a promising alternative substrate. CSW is rich in protein, chitin, and other nutrients, offering the potential for cost-effective and sustainable production of high-value products [24]. The use of KT2440 to ferment CSW for PHA production is a promising method to convert CSW into high-value waste, which not only enables the high-value conversion of CSW and the resourceful utilization of organic waste but also promises to reduce the cost of the substrate and solve the difficult problem of the high raw material cost in the production of PHA.

However, there is no reliable fermentation strategy for PHA production using CSW by KT2440. Meanwhile, limiting factors such as insufficient substrate protein utilization also inhibit the growth of microbial cells during fermentation, and high-nitrogen substrates limit the synthesis of the microbial intracellular product PHA [25]. How to explore reliable fermentation conditions through the rational modification of the protein utilization pathway, regulate the carbon and nitrogen balance in the metabolic process to effectively promote the growth and propagation of cells and improve the substrate yield has become a problem that needs to be solved. Therefore, this study aimed to systematically optimize the key fermentation parameters such as fermentation time, fermentation temperature, substrate solid–liquid ratio, and rotational speed for the fermentation of CSW by KT2440 through one-way and orthogonal experiments to improve the cell growth and synthesis of the product PHA. Meanwhile, combined with rational gene modification, it addresses the limiting factors of insufficient protein utilization of CSW substrate and high nitrogen substrate limitations, thus achieving improved cell growth and PHA conversion.

## 2. Results

### 2.1. Effect of Individual Fermentation Factors on the Maximum Growth of the Strain

#### 2.1.1. Time

**Figure 1:**
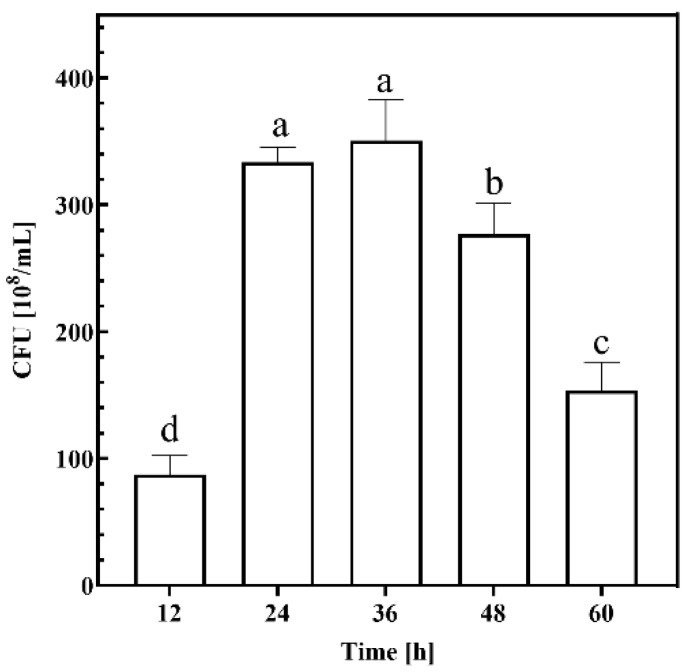
Growth performance of KT2440 at different experimental time points. Different lowercase letters indicate significant differences at *p* < 0.05 level.

To investigate the cell growth of KT2440 fermentation using CSW as substrate, the fermentation was set up with time as a single factor based on the initial conditions of substrate solid–liquid ratio of 5%, fermentation temperature of 30 °C, and speed of rotation of 200 rpm, and colony forming units (CFU) of the fermentation broth were measured after fermentation. The results are shown in Figure 1. At 12 h, the growth of the strain was at a low level, indicating that the growth of the strain at 12 h was still in the delayed or logarithmic phase, and the CFU reached a plateau at the time points of 24 h and 48 h. The highest level of CFU was reached at 36 h, reaching 350.67 × 10^8^/mL, which indicated that the growth and reproduction of KT2440 were the most active in this phase, and the CFU significantly decreased after the time point of 48 h, indicating that the growth of the strain was in the decay phase after 48 h. This shows that KT2440 is feasible to utilize substrate for cell growth when fermented in substrate CSW medium with a solid–liquid ratio of 5% at 30 °C and 200 rpm and that the cell growth plateau is in the range of about 24 h–48 h.

#### 2.1.2. Solid–Liquid Ratio

**Figure 2:**
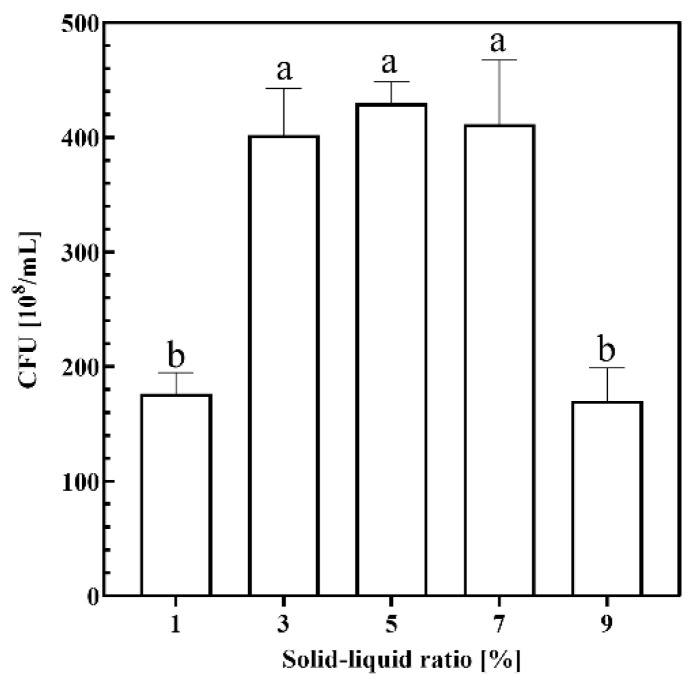
Growth performance of KT2440 in different substrate solid–liquid ratios. Different lowercase letters indicate significant differences at *p* < 0.05 level.

To investigate the effect of different substrate solid–liquid medium ratios on the growth of strain KT2440, fermentation was carried out using a CSW medium with different solid–liquid ratios as substrate and the fermentation broth CFU was determined. The results are shown in Figure 2, where CFU reached higher levels when the substrate solid–liquid ratio was in the range of 3% to 7%, indicating that the environmental conditions in this range of ratios were more suitable for the growth of KT2440. On the other hand, when the substrate solid–liquid ratio was 1% or 9%, the CFU was significantly reduced. It can be hypothesized that, when the substrate concentration was too low, it limited the growth of bacteria due to insufficient nutrients and intensified competition among bacteria, and that the accumulation of metabolites and substrate inhibition when the substrate concentration was too high limited the growth of KT2440 cells.

#### 2.1.3. Temperature

**Figure 3:**
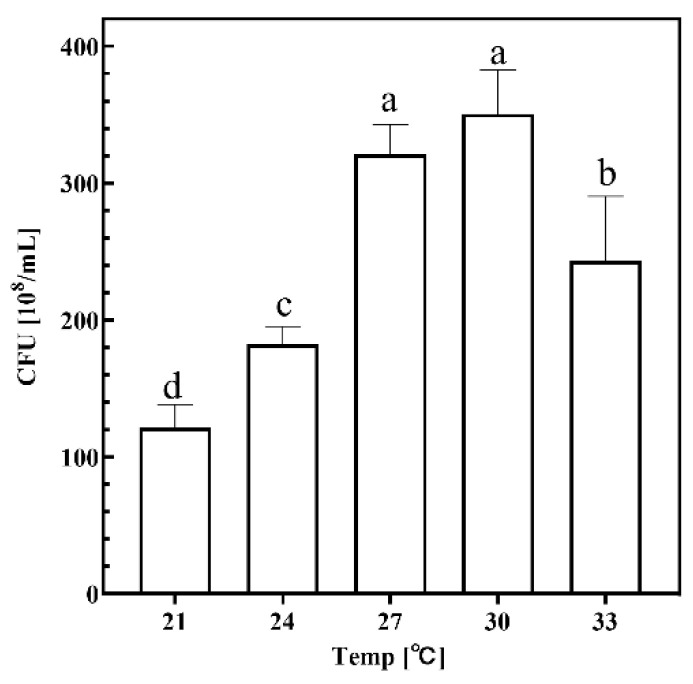
Growth performance of KT2440 in different temperatures. Different lowercase letters indicate significant differences at *p* < 0.05 level.

To investigate the effect of different temperatures on the growth of strain KT2440, the temperature of the thermostatic shaking incubator was set at different temperatures, and the CFU of the culture broth was measured after fermentation, and the results are shown in Figure 3. It can be seen that CFU reached a higher level in the temperature range of 27 °C and 30 °C, indicating that the growth and metabolism of KT2440 were the most vigorous in this temperature range and that the cell growth of KT2440 was affected by low (21 °C, 24 °C) and high (33 °C) temperatures with a significant decrease, indicating that temperature also has an important effect on the growth and metabolism of microorganisms, and the optimal temperature for cell growth was in the range of 27–30 °C.

#### 2.1.4. Rotation Speed

**Figure 4:**
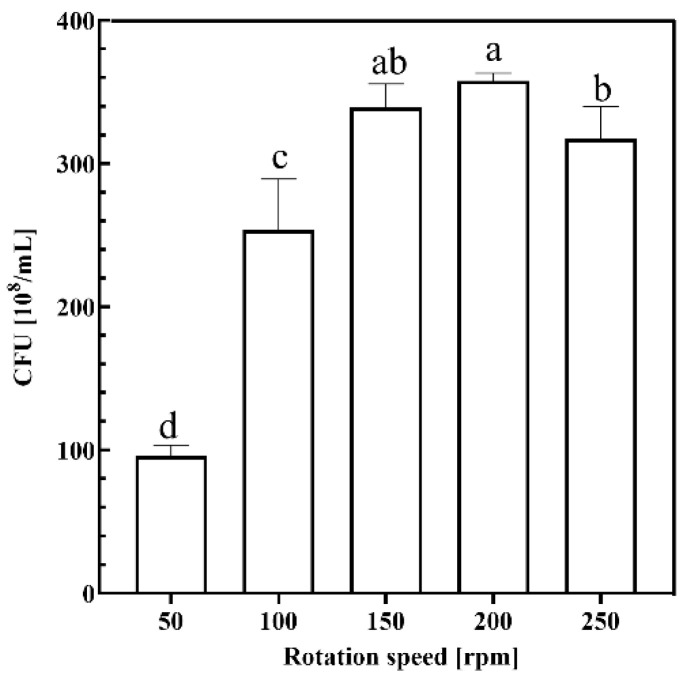
Growth performance of KT2440 in different rotation speeds. Different lowercase letters indicate significant differences at *p* < 0.05 level.

The effect of different fermentation rotational speeds on the growth of strain KT2440 is shown in Figure 4. The results showed that CFU reached a high level in the range of 150 rpm to 200 rpm, with 200 rpm being the highest level of 357.67 × 10^8^ /mL, indicating that the microorganisms grow in the most suitable environment in this speed range. On the other hand, cell growth was significantly reduced at rotational speeds below 150 rpm, indicating that too low a rotational speed may have resulted in reduced dissolved oxygen in the medium and insufficient contact between the substrate and the bacteria, thus affecting the efficiency of nutrient uptake by the strains, resulting in an increase in biomass but a prolongation of the growth cycle nonetheless. In addition, rotational speed that is too high (250 rpm) may cause damage to the cells, causing cell division to be blocked or die and resulting in low cell growth.

### 2.2. Optimal Fermentation Parameters Determined by Orthogonal Experiments

Based on the results of the single-factor test, a four-factor, three-level orthogonal test (See Section 4) was conducted with CFU as an indicator to further optimize the optimal process conditions for the fermentation of CSW by strain KT2440. With CFU as the primary indicator. As can be seen from Table 1, the highest CFU of 399 × 10⁸/mL was achieved under culture condition No. 5, while the lowest CFU of 176 × 10⁸/mL was observed under condition No. 1.

The influence of each parameter on CFU was evaluated by analyzing the extreme deviation (R-value) of the experimental results. The substrate solid–liquid ratio (parameter C) exhibited the largest R-value of 130.67, indicating that it had the most significant impact on CFU. This was followed by fermentation temperature (parameter A, R = 106.00), rotational speed (parameter D, R = 27.33), and fermentation time (parameter B, R = 5.67). These results demonstrate that the solid–liquid ratio and fermentation temperature are the most critical factors influencing CFU, while fermentation time has the least impact. The optimal combination of parameters was determined to be a fermentation temperature of 30 °C, fermentation time of 42 h, substrate solid–liquid ratio of 7%, and rotational speed of 200 rpm.

To further validate the significance of each parameter, an ANOVA analysis was performed. The results confirmed that the solid–liquid ratio (*p* < 0.01) and fermentation temperature (*p* < 0.05) had statistically significant effects on CFU, while the effects of rotational speed and fermentation time were less significant (*p* > 0.05). This supports the conclusion that the solid–liquid ratio and temperature are the primary factors driving microbial growth in the fermentation process.

### 2.3. Validation of Optimization of Fermentation Parameter Combinations

**Figure 5:**
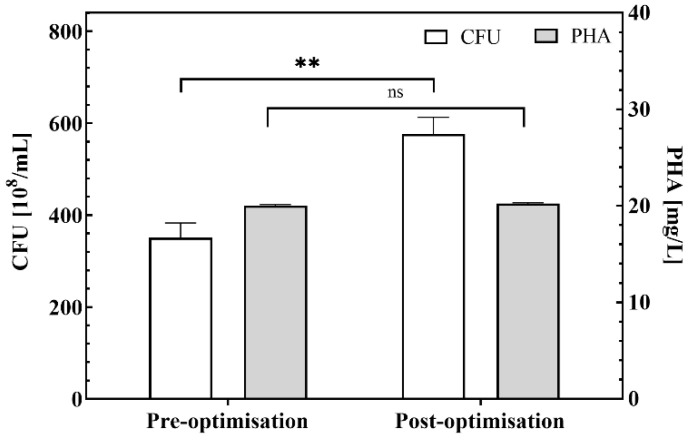
CFU and PHA yields at pre-optimization and post-optimization of fermentation parameters. ** *p* < 0.01 and ns not significant.

Optimized fermentation parameters (fermentation temperature 30 °C, fermentation time 42 h, substrate solid–liquid ratio 7%, rotational speed 200 rpm) obtained based on the combination of the above parameters, and the effect of fermentation parameters before optimization (fermentation temperature 30 °C, fermentation time 36 h, substrate solid–liquid ratio 5%, rotational speed 200 rpm) on cell growth and PHA synthesis are shown in Figure 5. After parameter optimization, the CFU increased from 350.67 × 10^8^ /mL to 576.44 × 10^8^ /mL, which was 64.39% higher and had a statistically significant difference. PHA content was 20.01 mg/L and 20.24 mg/L, respectively, with no significant difference. However, the ratio of PHA content to the value of CFU decreased by 2.22%, indicating that the PHA accumulation per unit cell decreased after optimization although the cell growth was significantly improved, and it was speculated that the increase in protein substrate content might have inhibited the accumulation of PHA.

### 2.4. Effects of Metabolic Engineering of Nitrogen Metabolism on Cell Growth and PHA Production

#### 2.4.1. Cell Growth

**Figure 6:**
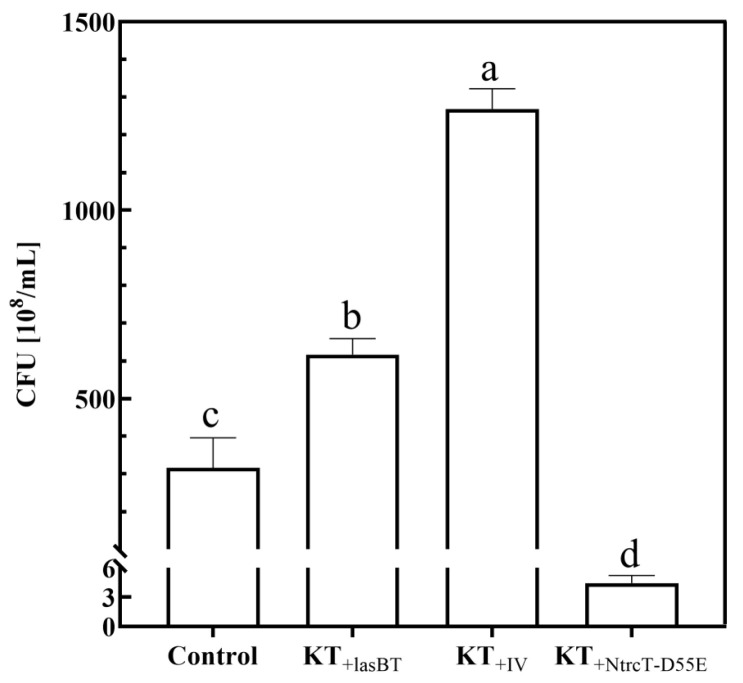
Protein regulatory gene overexpression strains fermented CFU. Different lowercase letters indicate significant differences at *p* < 0.05 level.

Based on these findings, the nitrogen metabolism pathway of KT2440 was modified to address the limiting factor of underutilization of substrate proteins during fermentation of CWS (there was no significant difference in cell growth between 3%, 5%, and 7% substrate solid–liquid ratios). After optimization, two engineered strains overexpressing two exogenous proteases: KT_+IV_ and KT_+lasBT_ were constructed and an engineered strain modifying the nitrogen metabolism regulatory gene (*ntrC*) in KT2440, KT_+NtrcT-D55E_ was constructed (See Section 4). To investigate the cell growth of engineering strains, the overexpression strains served as experimental groups, the KT_G_ strain was set as a control, and the maximum growth of the strains was determined using optimized culture parameters. The results are shown in Figure 6. Compared with the control group (316.44 × 10^8^ /mL), the cell growth of KT_+lasBT_ (616.89 × 10^8^ /mL) and KT_+IV_ (1268.44 × 10^8^ /mL) strains significantly increased by 94.94% and 300.84%, while the cell growth of KT_+NtrcT-D55E_ strain significantly decreased by 98.60%. It can be seen that overexpression of the *lasBT* gene and *IV* gene can significantly increase its cell growth, but the magnitude of the increase is different, indicating that the two exogenous proteases can play a role in KT2440, but there are differences in their functions. Overexpression of the *D55E* point mutant gene of homologous *ntrC* protein may have inhibited the N metabolic pathway of the cell and reduced cell growth.

#### 2.4.2. PHA Production

**Figure 7:**
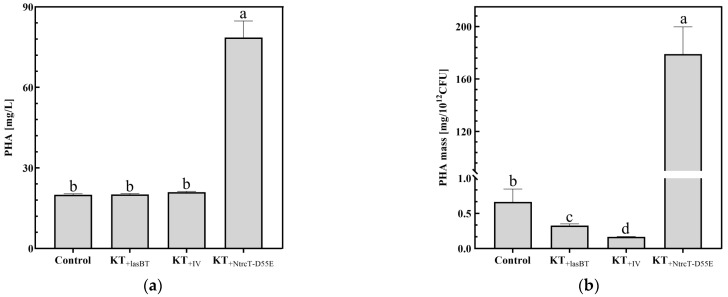
Protein regulatory gene overexpression strains fermented PHA. (**a**) PHA yield by different strains; (**b**) PHA accumulation per unit cell. Different lowercase letters indicate significant differences at *p* < 0.05 level.

To investigate the effect of overexpression of protein-regulated gene strains on PHA production in the fermentation of CSW, the overexpression strains were taken and fermented under optimized parameter conditions, and KT_G_ strains were set as the control group,) and the PHA accumulation in the cells was detected, and the results are shown in Figure 7a. From the PHA yield, it can be seen that the PHA yield of KT_+NtrcT-D55E_ reached 78.58 mg/L, which is a significant increase of 292.93% compared to the control (20.00 mg/L), while the yield of the other two strains remained almost unchanged. It can be inferred that the two strains obtained by overexpressing the exogenous protease instead decreased the amount of PHA accumulated per unit cell as cell growth increased; overexpression of the *D55E* point mutation gene of the homologous *ntrC* protein increased the strains not only to increase the cellular yield of PHA but also to increase the amount of PHA accumulated per unit cell (Figure 7b).

#### 2.4.3. PHA Monomer Composition Analysis

The composition of PHA is shown in Table 2. The results showed that the composition of PHA monomers from the wild-type strain and the engineered strain had the lowest content of C_6_, with a maximum of no more than 2.58%, and the highest content of C_10_, which could reach 44.75%. Compared with the wild-type strain and the control, there was no significant difference in the content of the other components of KT_+laSBT_ and KT_+IV_, except for the C_8_ component of KT_+IV_. Notably, compared with the wild-type and control strains, the C_12_ content of PHA polymer in KT_+NtrcT-D55E_ decreased significantly and was the lowest among the groups (27.83%), and the contents of C_8_ and C_10_ components both increased and were the highest among the groups, which were 24.63% and 44.75%, respectively, indicating that the main PHA polymer in the KT_+NtrcT-D55E_ unit shifted from a long carbon chain unit (C_12_) to a shorter carbon chain unit (C_8_, C_10_), speculating that different engineered strains may have affected the downstream metabolic pathways involved in the synthesis of PHA and changed the composition of the monomers.

## 3. Discussion

In this study, *P. putida* KT2440 was successfully screened and fermented with *Portunus trituberculatus* crab shell as the substrate, which could utilize the protein and other nutrients in CSW for growth and accumulate PHA intracellularly, which provided a new idea for *P. putida* to produce high-value bio-plastic PHA and high-value utilization of CSW. It has been pointed out that CSW is rich in proteins [2], chitin [3], and other nutrients, which can provide carbon and nitrogen sources for the growth of *Pseudomonas* and the synthesis of PHA during the fermentation process.

Fermentation conditions are the key elements in the fermentation process, which directly affects the growth and metabolism of microorganisms, and suitable conditions allow good growth of microorganisms. The effect of fermentation conditions on cell growth is one of the focuses of this study, and key parameters such as fermentation temperature, fermentation time, substrate solid–liquid ratio, and rotational speed were screened for their effects on CFU through single-factor and orthogonal experiments. The number of cell growth was significantly increased after optimization, indicating that the appropriate fermentation conditions are crucial for improving the cell growth and the yield of fermentation products. During the fermentation process, the number of microbial cells can increase the fermentation; when the fermentation time is too long, cell growth will be inhibited or even lead to cell death. Temperature can affect the activity of enzymes in microorganisms, which in turn affects their growth, reproduction, and metabolite synthesis [26]; The substrate solid–liquid ratio determines the microbial nutrient concentration and growth environment, affecting its growth and product synthesis and fermentation process stability [27]. The rotational speed affects the oxygen supply and nutrient transfer, as well as the morphology and enzyme activity of microorganisms; too low a rotational speed will make the microorganisms hypoxic and malnourished, while too high will lead to deformation of the bacterium and a decrease in enzyme activity [28].

In future practical applications, these parameters should be adjusted to optimize the production process based on actual economics, e.g., reducing fermentation time, a less influential fermentation parameter, to shorten the production time. Similar findings were reported by Piedade et al. [29], who demonstrated that the solid–liquid ratio and temperature are critical factors in optimizing fermentation processes for biomass conversion. Their work also highlighted the importance of minimizing unnecessary variables, such as fermentation time, to improve process efficiency. Meanwhile, other influencing factors or fermentation methods can be further investigated, such as Park et al. (2023) [30]. The production of PHA can be effectively enhanced by adopting a suitable replenishment batch strategy. In addition, this study illustrated that different fermentation conditions were able to affect cell growth, but, in general, did not have a significant effect on substrate consumption, a possible reason for this being the ability to increase the efficiency of substrate utilization when cell growth is positive [31]. Further studies can also draw on previous substrate treatment methods, e.g., Huang et al. (2022) [3] suggested that the use of a natural deep eutectic solvent (NADES) can effectively remove proteins and minerals from CSW, thus improving chitin yield and quality. Harkin. et al. (2015) [32] suggested that increasing chitin production from *Cancer pagurus* shell waste by screening bacteria with proteolytic enzymes and acid-producing capacity can facilitate protein and calcium carbonate removal. This can help to determine how to optimize the pretreatment of CSW to increase the utilization of nutrients in CSW by KT2440, thereby improving cell growth and product yield.

The advantages and application prospects of Pseudomonas were further demonstrated in this study. However, by changing the fermentation conditions of the wild-type strain KT2440 to ferment CSW, there was some increase in cell growth and PHA accumulation, but CFU only increased by 64.39% and PHA by 1.13%, while the ratio of PHA content to the value of CFU decreased by 2.22%, indicating that the accumulation of PHA per unit of cell was reduced. Possible reasons for this are that changing the fermentation conditions reduces the constraints on microbial growth, and the microorganisms use more energy and material to maintain their growth and other basic metabolic activities, resulting in a relative decrease in the amount of energy used to synthesize PHA, or that the activities of enzymes related to PHA synthesis are reduced by the change in fermentation conditions. This still leaves lots of room for improvement in the production of PHA using *Pseudomonas*-fermented CSW.

Therefore, the nitrogen metabolism pathway of KT2440 was modified in this study. Two engineered strains were constructed by overexpressing two exogenous proteases: KT_+IV_ and KT_+lasBT_; and an engineered strain, KT_+NtrcT-D55E_, was constructed by modifying the N metabolism regulatory gene (*ntrC*) of KT2440 itself. Hervas et al. (2009) [33] showed that *ntrC* is a transcriptional activator that plays a key role in nitrogen assimilation and is involved in the regulation of nitrogen metabolism by the *D55E* expression, a point mutation in the amino acid sequence of the *ntrC* protein in KT2440, which has a regulatory effect on nitrogen metabolism. Traidej et al. (2003) [34] investigated the expression of the protease *IV* gene in *Pseudomonas aeruginosa* and showed that *P. putida* expressing the protease *IV* gene had higher extracellular enzyme activity than *P. aeruginosa*. Thibodeaux et al. (2007) [35] studied the elastase B, or LasB protein of *Pseudomonas aeruginosa*, and showed that the *lasBT* gene encodes a related protein and can regulate the expression and secretion of related proteins. These findings suggest a wide range of regulation of the above protein genes.

The results of the fermentation of the overexpressed protein-regulated strains showed that the cell growth of the KT_+lasBT_ and KT_+IV_ strains significantly increased by 94.94% and 300.84%, while the cell growth of the KT_+NtrcT-D55E_ strain significantly decreased by 98.60% compared to the control group. Compared with the control, the PHA production of KT_+NtrcT-D55E_ was significantly increased by 292.93%, while the production of the other two strains remained almost unchanged. This suggests that upon overexpression of the point mutant *D55E* gene, which is homologous to the *ntrC* protein, the N-restriction condition allows KT2440 to optimize its metabolic pathways, reduce its cellular value-added, and use PHA as carbon and energy for intracellular storage. After overexpression of *IV* and *lasBT* genes, it increased the extracellular enzyme activity of KT2440 and promoted the role of biofilm formation-related proteins, which led to an increase in the efficiency of protein utilization, thus entering into a high nitrogen state and improving its cellular growth and differentiation, though there was no significant increase in the synthesis of PHA.

In subsequent studies, KT2440 can be genetically edited in conjunction with gene editing techniques to enable it to simultaneously enhance cell growth and metabolite synthesis, for example, by integrating the point mutation *D55E* gene and *IV* gene of homologous *ntrC* protein and *lasBT* gene. This resulted in the production of mutant strains that could simultaneously increase cell growth and product accumulation. Also, the results of Tan et al. (2020) [36] can be referenced to edit the PHA synthase gene cluster and modify the strain in terms of production of PHA monomer fractions to obtain a more targeted and high-value transformation. In addition, in the actual production, it can be combined with Harkin, C. et al. (2015) [37] in the treatment of *Cancer pagurus* shell waste, to reduce the production cost and improve the economic efficiency through the collection, treatment, utilization of CSW and other wastes from different perspectives as well as combining with related industries, and to assess the impact of this production process on the environment to ensure that it is sustainable, which is an important aspect to focus on in future research.

Furthermore, the economic feasibility of this study is highly promising, as it utilizes shrimp and crab shell waste (CSW) as a low-cost substrate for fermentation. This approach not only reduces raw material costs but also provides significant environmental benefits by converting waste into valuable bioproducts. As a proof-of-concept study, our research lays the foundation for future developments in sustainable PHA production. With further optimization and scaling, this method has the potential to become a cost-effective and environmentally friendly alternative to traditional PHA production processes, contributing to the circular economy and reducing the environmental footprint of plastic production.

## 4. Materials and Methods

### 4.1. Experimental Materials

#### 4.1.1. Crab Shell Preparation

To accurately replicate the actual production conditions, the experimental *Portunus trituberculatus* used for crab shell preparation was sourced from the Research Academy of Sanmen Mud Crab Industrial Technology, Taizhou City, in China. Upon delivery, the crabs were steamed at 105 °C for 15 min, then cooled to room temperature. The muscles and internal organs were carefully removed using dissection tools, while the shell was retained. The shells were then washed with water, dried at a constant temperature of 55 °C for 12 h, and subsequently crushed in a mortar to a 4-mesh consistency. Finally, the crushed material was dried at a constant temperature of 55 °C until reaching a constant weight.

#### 4.1.2. Culture Media

In this study, the medium was prepared using the following method: A solution consisting of 10 g/L peptone, 5 g/L yeast extract, and 5 g/L sodium chloride was autoclaved at 121 °C for 20 min, then cooled to create the LB liquid medium. For the LB agar medium, a combination of 10 g/L peptone, 5 g/L yeast extract, 5 g/L sodium chloride, and 15 g/L agar was dissolved in deionized water. This solution was autoclaved at 121 °C for 20 min, cooled to 60 °C, thoroughly shaken, and then poured into plates to solidify. The substrate utilized was a crab shell culture medium derived from *Portunus trituberculatus*, prepared by adding crushed crab shells to 250 mL conical flasks, with the mass of the shells determined according to the solid–liquid ratio. Following the addition of 100 mL of deionized water, the mixture was shaken well, and the flasks were sealed with a film before being autoclaved at 121 °C for 20 min. The growth condition of KT2440 was determined by counting CFU, and CFU was determined by the dilution spread plate method. Briefly, 100 μL of the 10^−7^-fold dilution was spread uniformly on LB agar medium and incubated for 36 h at 30 °C in a thermostatic incubator, and the number of colony growths was counted.

#### 4.1.3. Plasmids and Strains Construction

The plasmids and bacterial strains used in this study are listed in Table 3. Primers used for vector construction are listed in Table 4. The *E. coli* DH5α strain was used for all molecular manipulations during plasmid construction [38] and was cultured on LB medium at 37 °C.

The plasmids were constructed according to the standard molecular cloning protocol and were obtained by enzymatic ligation. The sequences were synthetic constructs, optimized according to KT2440 codon preference, and uploaded to NCBI with gene accession numbers PQ788834 for Ntrc-D55E, PQ788835 for lasBT, and PQ788836 for IV, respectively. Taking the construction of the plasmid vector pPR-NtrcT-D55E as an example (the rest of the vector process is the same), the NtrcT-D55E fragment was obtained by PCR amplification using the primers D55E F61/D55E R61 and the high-fidelity enzyme 2×Prime STAR^®^ Mix, and the purified fragment was obtained by recovery. The pUCP18-Gm plasmid vector and the NtrcT-D55E fragment were double digested with restriction endonucleases BamHI and SacI, incubated at 37 °C for 3–4 h, and the gel was cut and recovered to obtain the purified backbone fragment and insert fragment. The plasmid backbone and insert fragments were ligated using T4 DNA ligase at a molar ratio of 1:3 and ligated overnight at 16 °C in a PCR instrument (Thermo Fisher Scientific, Waltham, MA, USA).

**Figure 8:**
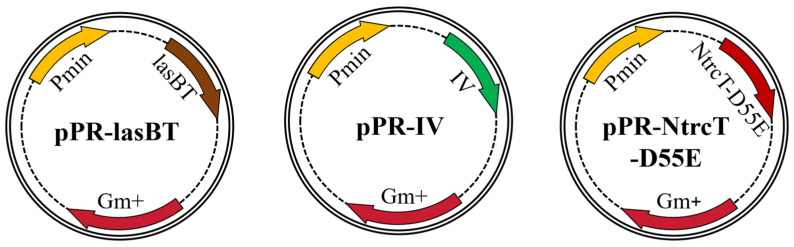
Schematic diagram of overexpression vectors for protein-regulated genes.

The overexpression plasmid vector was transformed into *E. coli* DH5α and then screened on LB agar supplemented with 30 μg/mL gentamicin; the transformants were verified by PCR and plasmid was extracted for enzymatic verification and sequencing. A wild-type KT2440 strain was used as the initial strain. Three engineered strains, KT_+NtrcT-D55E_, KT_+IV_, and KT_+lasBT_ (overexpression vectors shown in Figure 8), were obtained by electroporation (2.5 kv, 200 Ω) of validated and relevant plasmids into KT2440 by using a gene importer (Scientz-2C, China).

### 4.2. Inoculation and Fermentation

For the LB liquid medium and the crab shell waste substrate, a 1% volume ratio for inoculation was used. In a sterile ultra-clean bench, 1% *v*/*v* of the seed solution was taken and transferred to the inoculation medium. The container was sealed, shaken thoroughly, and the fermentation was incubated in a temperature-controlled shaker. Using genetically modified *Pseudomonas putida* KT2440 may raise important regulatory and environmental issues. To minimize the risk, we have implemented stringent biosafety measures, including physical isolation and the use of nutrient-deficient strains to prevent accidental spread. All were inactivated upon completion of fermentation.

### 4.3. Single Factor of Fermentation

To explore the feasibility and optimal fermentation conditions for KT2440 in the production of high-value chemicals, such as PHA, through fermentation utilizing CWS as a substrate, this study examined the effects of four key factors on the strain’s growth. These factors included time, solid–liquid ratio, temperature, and rotational speed. Each group was set with five levels, based on the initial fermentation conditions of 36 h, a substrate solid–liquid ratio of 5%, a fermentation temperature of 30 °C, and a rotational speed of 200 rpm.

In the time group, fermentation was halted at 12 h, 24 h, 36 h, 48 h, and 60 h, with the CFU of the fermentation broth measured at each interval. For the solid–liquid ratio group, fermentation was conducted using crab shell medium at solid–liquid ratios of 1%, 3%, 5%, 7%, and 9%, and the CFU of the fermentation broth was subsequently determined. In the temperature group, the thermostatic oscillation incubator was set to temperatures of 21 °C, 24 °C, 27 °C, 30 °C, and 33 °C, followed by measurement of the CFU in the culture broth after fermentation. In the rotational speed group, the incubator’s rotational speed was adjusted to 50 rpm, 100 rpm, 150 rpm, 200 rpm, and 250 rpm for fermentation, with CFU measurements taken post-fermentation.

### 4.4. Orthogonal Experiments to Optimize Fermentation Parameter Combinations

Based on the results of the one-way experiment, an L_9_ (3^4^) orthogonal experiment was designed for fermentation using CFU as a reference index for four important fermentation parameters: fermentation time, substrate solid–liquid ratio, fermentation temperature, and rotational speed. The designed parameters and their levels are shown in Table 5. The CFU of the culture broth was measured after fermentation.

### 4.5. Measurement of PHA Content

For PHA extraction and content determination, refer to Lin et al. (2016) [40] and Zhou et al. (2024) [41]. After fermentation and cultivation of the strain, the bacterial liquid was obtained by filtration through a 200-mesh gauze silk; the bottom bacterial body was collected by centrifugation, placed in the refrigerator at −80 °C for 12 h, and then freeze-dried to obtain the dried cell powder. mcl-PHA was extracted from the freeze-dried cell samples using the hot chloroform method and purified by centrifugation with methanol.

For PHA yield and compositional analyses, each purified mcl-PHA was esterified and solubilized by 50-fold dilution with hexane and determined using gas chromatography-tandem mass spectrometry (GC-MS; Agilent, Santa Clara, CA, USA) according to established protocols. An Agilent micro-sampler was used with an injection volume of 1 μL and a carrier gas of helium at a flow rate of 1 mL/min. The inlet temperature was 250 °C, the injection mode was non-split, the injection time was 1 min, and the starting temperature of the injection box was 100 °C, held for 1 min, and then warmed up from 100 °C to 280 °C at a rate of 30 °C/min, held for 5 min. The C6, C8, C10, and C12 standards were mixed to create a standard curve with a detection range of 5–25 ppm. The C6 standard curve is y = 80,441x − 5846.2, R^2^ = 0.98; the C8 standard curve is y = 82,735x – 139,808, R^2^ = 0.99; the C10 standard curve is y = 109,737x – 343,492, R^2^ = 0.98; and the C12 standard curve is y = 67,125x – 194,372, R^2^ = 0.98. The peak area calculation method was employed to determine the relative content of each component within the PHA.

### 4.6. Data Processing

Data in this experiment were analyzed by one-way ANOVA and Tukey’s HSD test (*p* < 0.05) using SPSS Statistics 17.0 software, and graphs were plotted by Graphpad Pism 9.5 software. In addition, the results of orthogonal experiments were analyzed using Orthogonal Design Assistant II 3.1 software. Each experiment was repeated three times, and the results were expressed as mean values ± SD.

## 5. Conclusions

In summary, this study provides an important theoretical and experimental basis for the biosynthesis of high-value bioplastics using CSW substrate by KT2440. Firstly, the key parameters such as fermentation time, fermentation temperature, substrate solid–liquid ratio, and rotational speed were systematically optimized by single factor and orthogonal experiments, and the fermentation conditions for PHA production using KT2440 fermented CSW were explored. Then, by metabolic engineering of *P. putida* KT2440, two engineered strains, KT_+IV_ and KT_+lasBT_, which expressed exogenous proteases that could significantly enhance cell growth, were obtained; at the same time, an engineered strain, KT_+NtrcT-D55E_, which regulated nitrogen metabolism, was obtained, and the intracellular PHA accumulation significantly increased. Considering the relatively higher PHA yield in contrast with the considerably lower cell growth, future research will focus on the balance of cell growth and PHA yield with further exploration of nitrogen metabolism and PHA synthase pathway. Collectively, this study promotes the high-value conversion of crab shell waste, providing a new strategy for the synthesis of various high-value bioproducts through CSW fermentation.

## Figures and Tables

**Table 1:** Results and analysis of orthogonal experiments.

Experiment Number	A	B	C	D	
	Temp (°C)	Time (h)	Solid/Liquid Ratio (%)	Rotation Speed (rpm)	CFU (10^8^/mL)
1	1 (27 °C)	1 (30 h)	1 (3%)	1 (150 rpm)	176
2	1	2 (36 h)	2 (5%)	2 (200 rpm)	301
3	1	3 (42 h)	3 (7%)	3 (250 rpm)	337
4	2 (30 °C)	1	2	3	386
5	2	2	3	1	399
6	2	3	1	2	299
7	3 (33 °C)	1	3	2	318
8	3	2	1	3	187
9	3	3	2	1	261
K1	814	880	662	836	
K2	1084	887	948	918	
K3	766	897	1054	910	
k1	271.33	293.33	220.67	278.67	
k2	361.33	295.67	316.00	306.00	
k3	255.33	299.00	351.33	303.33	
R	106.00	5.67	130.67	27.33	
*p*-value	*p* < 0.05	*p* > 0.05	*p* < 0.01	*p* > 0.05	
Priority of factors	C > A > D > B				
Optimal composition	A_2_B_3_C_3_D_2_				

**Table 2:** Analysis of the GC composition of PHA produced by strains overexpressing protein regulatory genes.

Monomer	PHA Composition (mol%)					
	WT	Control	KT_+lasBT_	KT_+IV_	KT_+NtrcT-D55E_	*p*-Value
3HHx (C_6_)	1.15 ± 0.05	1.64 ± 0.35	2.58 ± 0.90	1.34 ± 0.04	2.79 ± 0.02	0.077
3HO (C_8_)	21.98 ± 0.18 ^c^	21.67 ± 0.27 ^c^	21.61 ± 0.19 ^c^	23.09 ± 0.16 ^b^	24.63 ± 0.08 ^a^	0.000
3HD (C_10_)	40.18 ± 0.08 ^b^	39.93 ± 0.16 ^b^	39.55 ± 0.36 ^b^	39.92 ± 0.04 ^b^	44.75 ± 0.75 ^a^	0.000
3HDD (C_12_)	36.69 ± 0.21 ^a^	36.76 ± 0.09 ^a^	36.26 ± 0.36 ^a^	35.66 ± 0.16 ^a^	27.83 ± 0.81 ^b^	0.000

3HHx, 3-hydroxyhexanoate; 3HO, 3-hydroxyoctanoate; 3HD, 3-hydroxydecanoate; 3HDD, 3-hydroxydodecanoate; 3HTD, 3-hydroxytetradecanoate. Different lowercase letters indicate significant differences at *p* < 0.05 level.

**Table 3:** Plasmids and bacterial strains were used in this study.

Plasmid Vectors/Strains	Plasmid/Strain Characteristics	Source
Plasmid vector		
pUCP18-Gm	Ampr; Gmr, shuttle vector; lacZ with MCS	Cao et al. [39]
pPR-NtrcT-D55E	pUCP18-Gm derivative, P_min_:: NtrcT-D55E	This study
pPR-IV	pUCP18-Gm derivative, P_min_:: IV	This study
pPR-lasBT	pUCP18-Gm derivative, P_min_:: *lasBT*	This study
Strains		
*P. putida* KT2440	Wild type	Lab stock
KT_G_ (control strain)	KT2440 carrying pUCP18-Gm	This study
KT_+NtrcT-D55E_	KT2440 carrying pPR-NtrcT-D55E	This study
KT_+IV_	KT2440 carrying pPR-IV	This study
KT_+lasBT_	KT2440 carrying pPR-lasBT	This study

**Table 4:** Primers were used for vector construction in this study.

Primer Name	Sequence (5′-3′)
D55E F61	tggtaaagagctcatgagccgaagtg
D55E R61	cacctcagtggtcatcaccttcctc
IV F	cggaattccatgcataagagaacgtacctgaat
IV R	ggatcctcagggcgcgaagtagcgggagat
lasBT F	ccgcggatccaaataaaacgaaaggctcagtcg
lasBT R	cccggaattcaaaaggccatccgtcaggat

**Table 5:** Orthogonal experimental design.

Level	A	B	C	D
	Temp (°C)	Time (h)	Solid/Liquid Ratio (%)	Rotation Speed (rpm)
1	27	30	3	150
2	30	36	5	200
3	33	42	7	250

## Data Availability

The raw data supporting the conclusions of this article will be made available by the authors on request.

## References

[1] Lipp M., Bessy C., Cannavan A., Dupouy E., Fattori V., Kopko C., Lejeune J., Mukherjee K., Ferreira J.P., Schulz D., Smithers G.W. (2024). Food and Agriculture Organization of the United Nations (FAO). Encyclopedia of Food Safety (Second Edition).

[2] Nekvapil F., Ganea I.V., Ciorita A., Hirian R., Ogresta L., Glamuzina B., Roba C., Pinzaru S.C. (2021). Wasted Biomaterials from Crustaceans as a Compliant Natural Product Regarding Microbiological, Antibacterial Properties and Heavy Metal Content for Reuse in Blue Bioeconomy: A Preliminary Study. J. Mater..

[3] Huang W.C., Zhao D., Xue C., Mao X. (2022). An Efficient Method for Chitin Production from Crab Shells by a Natural Deep Eutectic Solvent. Mar. Life Sci. Technol..

[4] Sugiyanti D., Darmadji P., Anggrahini S., Anwar C., Santoso U. (2018). Preparation and Characterization of Chitosan from Indonesian Tambak Lorok Shrimp Shell Waste and Crab Shell Waste. J. Pak. J. Nutr..

[5] Zang B., Wang Y., Wang G., Zang Q., Chen H., Liu B. (2020). A Comparative Study on the Yields of Natural Astaxanthin Esters Extracted from Shrimp Shells Using Four Different Organic Solvents. Zhejiang Chem. Ind..

[6] Drakonaki A., Mathioudaki E., Geladas E.D., Konsolaki E., Vitsaxakis N., Chaniotakis N., Xie H., Tsiotis G. (2023). Production of Polyhydroxybutyrate by Genetically Modified *Pseudomonas* sp. phDV1: A Comparative Study of Utilizing Wine Industry Waste as a Carbon Source. Microorganisms.

[7] Wang Y., Wilks J.C., Danhorn T., Ramos I., Croal L., Newman D.K. (2011). Phenazine-1-Carboxylic Acid Promotes Bacterial Biofilm Development via Ferrous Iron Acquisition. J. Bacteriol..

[8] Letzel A.C., Pidot S.J., Hertweck C. (2014). Genome Mining for Ribosomally Synthesized and Post-Translationally Modified Peptides (RiPPs) in Anaerobic Bacteria. BMC Genom..

[9] Jyot J., Balloy V., Jouvion G., Verma A., Touqui L., Huerre M., Chignard M., Ramphal R. (2011). Type II Secretion System of *Pseudomonas Aeruginosa*: In Vivo Evidence of a Significant Role in Death Due to Lung Infection. J. Infect. Dis..

[10] Reddy M.V., Nikhil G.N., Mohan S.V., Swamy Y.V., Sarma P.N. (2012). Pseudomonas Otitidis as a Potential Biocatalyst for Polyhydroxyalkanoates (PHA) Synthesis Using Synthetic Wastewater and Acidogenic Effluents. Bioresour. Technol..

[11] Koller M., Bona R., Chiellini E., Fernandes E.G., Horvat P., Kutschera C., Hesse P., Braunegg G. (2008). Polyhydroxyalkanoate Production from Whey by Pseudomonas Hydrogenovora. Bioresour. Technol..

[12] Wongsirichot P., Gonzalez-Miquel M., Winterburn J. (2020). Integrated Biorefining Approach for the Production of Polyhydroxyalkanoates from Enzymatically Hydrolyzed Rapeseed Meal under Nitrogen-Limited Conditions. ACS Sustain. Chem. Eng..

[13] Wang Q., Nomura C.T. (2010). Monitoring Differences in Gene Expression Levels and Polyhydroxyalkanoate (PHA) Production in Pseudomonas Putida KT2440 Grown on Different Carbon Sources. J. Biosci. Bioeng..

[14] Torrego-Solana N., Martin-Arjol I., Bassas-Galia M., Diaz P., Manresa A. (2012). Hydroxy-Fatty Acid Production in a Pseudomonas Aeruginosa 42A2 PHA Synthase Mutant Generated by Directed Mutagenesis. Appl. Microb. Biotechnol..

[15] Hori K., Ichinohe R., Unno H., Marsudi S. (2011). Simultaneous Syntheses of Polyhydroxyalkanoates and Rhamnolipids by *Pseudomonas aeruginosa* IFO3924 at Various Temperatures and from Various Fatty Acids. Biochem. Eng. J..

[16] Chen J.Y., Song G., Chen G.Q. (2006). A Lower Specificity PhaC2 Synthase from *Pseudomonas stutzeri* Catalyses the Production of Copolyesters Consisting of Short-Chain-Length and Medium-Chain-Length 3-Hydroxyalkanoates. Antonie Van Leeuwenhoek.

[17] Martinez-Garcia E., de Lorenzo V. (2011). Engineering Multiple Genomic Deletions in Gram-Negative Bacteria: Analysis of the Multi-Resistant Antibiotic Profile of *Pseudomonas putida* KT2440. Environ. Microbiol..

[18] Wang S., Cui J., Bilal M., Hu H., Wang W., Zhang X. (2020). *Pseudomonas* spp. as Cell Factories (MCFs) for Value-Added Products: From Rational Design to Industrial Applications. Crit. Rev. Biotechnol..

[19] Mitra R., Xu T., Xiang H., Han J. (2020). Current Developments on Polyhydroxyalkanoates Synthesis by Using Halophiles as a Promising Cell Factory. Microb Cell Fact..

[20] Chen G.Q., Jiang X.-R., Guo Y. (2016). Synthetic Biology of Microbes Synthesizing Polyhydroxyalkanoates (PHA). Synth. Syst. Biotechnol..

[21] Lim J., You M., Li J., Li Z. (2017). Emerging Bone Tissue Engineering via Polyhydroxyalkanoate (PHA)-Based Scaffolds. Mater. Sci. Eng. C.

[22] Costa S.G.V.A.O., Lepine F., Milot S., Deziel E., Nitschke M., Contiero J. (2009). Cassava Wastewater as a Substrate for the Simultaneous Production of Rhamnolipids and Polyhydroxyalkanoates by *Pseudomonas aeruginosa*. J. Ind. Microbiol. Biotechnol..

[23] Kosseva M.R., Rusbandi E. (2018). Trends in the Biomanufacture of Polyhydroxyalkanoates with Focus on Downstream Processing. Int. J. Biol. Macromol..

[24] Komilus C.F., Mohamad-Zuki N.A., Aminudin N.H., Redhwan A.I., Mohd-Khairulnizam N.A.N. (2023). Effects of Crab Shell Waste as Feed on Growth Performance and Colouration of Siamese Fighting Fish (Betta Splendens). Malays. Appl. Biol..

[25] Titz M., Kettl K.H., Shahzad K., Koller M., Schnitzer H., Narodoslawsky M. (2012). Process Optimization for Efficient Biomediated PHA Production from Animal-Based Waste Streams. Clean Technol. Environ. Policy.

[26] Darken M.A., Berenson H., Shirk R.J., Sjolander N.O. (1960). Production of Tetracycline by *Streptomyces aureofaciens* in Synthetic Media. Appl. Microbiol..

[27] Shanmugaprakash M., Vinoth Kumar V., Hemalatha M., Melbia V., Karthik P. (2011). Solid-state Fermentation for the Production of Debittering Enzyme Naringinase Using *Aspergillus niger* MTCC 1344. Eng. Life Sci..

[28] Huang B., Li D.-G., Huang Y., Liu C. (2018). Effects of Spaceflight and Simulated Microgravity on Microbial Growth and Secondary Metabolism. Mil. Med. Res..

[29] Piedade P.J., Venkat V., Al-Shwafy K.W.A., Aregawi M.A., Dudek G., Zygadło M., Lukasik R.M. (2024). Comprehensive Wheat Straw Processing with Deep Eutectic Solvent to Deliver Reducing Sugar. Bioenerg. Res..

[30] Park Y., Jeon J.-M., Park J.K., Yang Y.-H., Choi S.S., Yoon J.-J. (2023). Optimization of Polyhydroxyalkanoate Production in *Halomonas* Sp. YLGW01 Using Mixed Volatile Fatty Acids: A Study on Mixture Analysis and Fed-Batch Strategy. Microb. Cell Factories.

[31] Brown R.B., Klaus D., Todd P. (2002). Effects of Space Flight, Clinorotation, and Centrifugation on the Substrate Utilization Efficiency of *E. Coli*. Microgravity Sci. Technol..

[32] Xun J., Zhang X., Guo S., Lu H., Chen J. (2021). Editing out HIV: Application of Gene Editing Technology to Achieve Functional Cure. Retrovirology.

[33] Hervas A.B., Canosa I., Little R., Dixon R., Santero E. (2009). *NtrC*-Dependent Regulatory Network for Nitrogen Assimilation in *Pseudomonas putida*. J. Bacteriol..

[34] Traidej M., Caballero A.R., Marquart M.E., Thibodeaux B.A., O’Callaghan R.J. (2003). Molecular Analysis of *Pseudomonas aeruginosa* Protease *IV* Expressed in *Pseudomonas putida*. Investig. Ophthalmol. Vis. Sci..

[35] Thibodeaux B.A., Caballero A.R., Marquart M.E., Tommassen J., O’Callaghan R.J. (2007). Corneal Virulence of *Pseudomonas aeruginosa* Elastase B and Alkaline Protease Produced by *Pseudomonas putida*. Curr. Eye Res..

[36] Tan I.K.P., Foong C.P., Tan H.T., Lim H., Zain N.-A.A., Tan Y.C., Hoh C.C., Sudesh K. (2020). Polyhydroxyalkanoate (PHA) Synthase Genes and PHA-Associated Gene Clusters in *Pseudomonas* Spp. and *Janthinobacterium* spp. Isolated from Antarctica. J. Biotechnol..

[37] Cong L., Ran F.A., Cox D., Lin S., Barretto R., Habib N., Hsu P.D., Wu X., Jiang W., Marraffini L.A. (2013). Multiplex Genome Engineering Using CRISPR/Cas Systems. Science.

[38] Green M.R., Sambrook J. (2001). Molecular Cloning: A Laboratory Manual. Anal. Anal. Biochem..

[39] Cao L., Lin L., Sui H., Wang H., Zhang Z., Jiao N., Zhou J. (2021). Efficient Extracellular Laccase Secretion via Bio-Designed Secretory Apparatuses to Enhance Bacterial Utilization of Recalcitrant Lignin. Green Chem..

[40] Lin L., Cheng Y., Pu Y., Sun S., Li X., Jin M., Pierson E.A., Gross D.C., Dale B.E., Dai S.Y. (2016). Systems Biology-Guided Biodesign of Consolidated Lignin Conversion. Green Chem..

[41] Zhou Y., Zhang X., Yu W., Fu Y., Ni L., Yu J., Wang X., Song W., Wang C. (2024). Enhancing Pseudomonas Cell Growth for the Production of Medium-Chain-Length Polyhydroxyalkanoates from Antarctic Krill Shell Waste. Int. J. Biol. Macromol..

